# Shiga Toxin-Producing *Escherichia coli* Infections during Pregnancy

**DOI:** 10.3390/microorganisms6040111

**Published:** 2018-10-23

**Authors:** Flavia Sacerdoti, María Luján Scalise, Juliana Burdet, María Marta Amaral, Ana María Franchi, Cristina Ibarra

**Affiliations:** 1 Laboratorio de Fisiopatogenia, Instituto de Fisiología y Biofísica Bernardo Houssay (IFIBIO Houssay-CONICET), Departamento de Fisiología, Facultad de Medicina, Universidad de Buenos Aires, Paraguay 2155, Buenos Aires 1121, Argentina; lujan.scalise@gmail.com (M.L.S.); mmamaral74@gmail.com (M.M.A.); 2 Laboratorio de Hematología, Hospital Universitario Austral, Pilar, Buenos Aires 1629, Argentina; julianaburdet@yahoo.com.ar; 3 CEFYBO-CONICET, Universidad de Buenos Aires, Buenos Aires 1121, Argentina; anafranchi2000@gmail.com

**Keywords:** STEC infections, pregnancy, Shiga toxin, prevention

## Abstract

Gastrointestinal infection with Shiga toxin-producing *Escherichia coli* (STEC) causes diarrhea, hemorrhagic colitis, and hemolytic uremic syndrome (HUS), characterized by hemolytic anemia, thrombocytopenia and acute renal failure. The main virulence factor of STEC is Shiga toxin (Stx), which is responsible for HUS development. STEC can produce Stx type 1 and/or 2 (Stx1, Stx2) and their variants, Stx2 being more frequently associated with severe cases of HUS. This pathology occurs in 5–15% of cases with STEC infection when Stx gain access to the bloodstream and causes damage in the target organs such as the kidney and brain. STEC infections affect mainly young children, although the large HUS outbreak with a new Stx2-producing STEC O104:H4 in Europe in 2011 involved more adults than children, and women were over-represented. Maternal infections during pregnancy are associated with adverse pregnancy outcomes. Studies in rats showed that Stx2 binds to the utero-placental unit and causes adverse pregnancy outcomes. In this article, we provide a brief overview of Stx2 action on placental tissues and discuss whether they might cause pregnancy loss or preterm birth.

## 1. Introduction

Shiga toxin-producing *Escherichia coli* (STEC) cause a significant public health risk due to contamination of food and water supplies. STEC produces gastrointestinal infections that may produce diarrhea and hemorrhagic colitis, and is the principal cause of hemolytic uremic syndrome (HUS), a systemic complication that is attributed to the action of Shiga toxins (Stx) [1]. STEC can produce two antigenically diverse forms of Stx proteins (Stx1 and Stx2) and their variants, Stx2 being more virulent and epidemiologically more relevant than Stx1 [2]. Once ingested, STEC colonizes the human intestine, releasing Stx which crosses the epithelial barrier and reaches the target organs by the systemic circulation. It is well known that Stx binds Gb3 receptors on the endothelial cells, goes through receptor-mediated internalization followed by retrograde transport to the Golgi apparatus, and finally induces cell death by inhibition of protein synthesis and consequent cellular apoptosis [3].

HUS is characterized by thrombocytopenia, hemolytic anemia and acute renal failure. In Argentina, HUS is recognized as the most common cause of acute renal failure in children and the second leading cause of chronic renal failure [4,5]. In Argentina, HUS is endemic and has the highest rate of pediatric cases globally over the last 10 years, with approximately 400 HUS cases reported annually. This results in an incidence of 10–17 cases per 100,000 children less than 5 years of age, and lethality of 1 and 4% [6]. Although multiple serotypes of STEC have been isolated from hemorrhagic colitis cases, *E. coli* O157:H7 is the most prevalent serotype associated with HUS in children. However, the large HUS outbreak in 2011 in central Europe caused by a novel Stx2-producing STEC, affected more adults than children and women were over-represented [7], likely due to children not being eager consumers of fenugreek sprouts.

During pregnancy the maternal immune response drives physiological adaptations to tolerate the foreign fetus. However, many pathophysiological situations can interrupt the normal progression of pregnancy, including genetic and endocrinologic anomalies such as defects on invasion of trophoblast leading to alterations in the blood irrigation. Nevertheless, many etiologies of obstetric complication and pregnancy loss are unknown or poorly understood. Infections during pregnancy have been associated with higher incidence of spontaneous abortion, preterm birth or placental dysfunction [8,9]. However, to our knowledge, an increased risk of spontaneous abortion or preterm delivery in humans linked to STEC infection has not yet been evaluated.

## 2. Foodborne Bacteria and Adverse Pregnancy Outcome

It is estimated that infections are responsible for 10–25% of fetal deaths in developed countries, with bacterial infections more frequently associated with adverse outcomes during early gestation compared with late gestation [10]. Bacterial infections are especially feared because they may endanger not only the mother but also her child. Some infections take a more severe course during pregnancy, probably driven by the physiological and immune alterations in this period that induce an increased susceptibility to certain pathogens, including viruses, parasites and bacteria. Although immunologic changes during pregnancy may provide different conditions for entrance of certain infections, pregnant women are not considered to be immune-suppressed in the classic sense [11]. However, changes occurring at the feto-maternal interface in order to tolerate the fetal alloantigen may predispose pregnant women to infections [12]. Taking this into account, pregnant women are considered an at-risk group for infection caused by foodborne pathogens and often suffer more sequelae as a result of infection [13,14].

Foodborne disease is defined as any illness related to food ingestion or caused by an infectious agent carried by food. In some cases of foodborne infection, a pregnant woman may not feel sick but may still pass the illness to the fetus [15]. For instance, untreated infections may cause stillbirth, preterm labor or miscarriage by mechanisms including direct fetal infection, placental damage, and severe maternal illness. The fetal period is of great plasticity, and factors like maternal nutrition, hormones, and microbial insults can produce epigenetic changes (DNA methylations, for example) that may modify gene expression in the child, resulting in predisposition to impaired health in adulthood [16,17,18]. Generally, foodborne pathogens and their virulence factors are transmitted by the hematogenous route, indeed, systemic infection of the mother may result in the following events: septic abortion, sepsis causing premature birth or fetal death. The general mechanisms proposed for bacteria gaining access to the feto-maternal unit are: (a) hematogenous dissemination (transplacental infection), (b) ascension from the vagina and cervix, (c) accidental introduction at the time of invasive procedures such as amniocentesis, (d) chorionic villous (placental tissue) sampling or shunting for prenatal genetic diagnosis, (e) retrograde from the peritoneal cavity through the fallopian tubes, and (f) contaminated food or mouth infections [19,20]. Another essential point is that mother to child transmission of infection can occur not only during pregnancy (vertical transmission), but also during labour or in the postpartum period.

The effects of several human foodborne bacteria have been recognized for many years. Indeed, *Listeria monocytogenes*, *Coxiella burnetii*, *Campylobacter jejuni*, *Salmonella typhi*, and *E. coli* are well known foodborne bacterial pathogens that may produce adverse outcomes during pregnancy [21,22]. *Listeria monocytogens* is the most studied pathogen particularly affecting pregnancy, and it is responsible for long term consequences, mainly for the baby. Infection is transmitted by animals or animal products, usually milk and dairy products [23]. Thus, listeriosis is considered a zoonosis and has been implicated in stillbirth, preterm labor, newborn sepsis, and meningitis, among other complications [14]. Even if the effects of some infectious bacterial agents are well known, currently there is still a lack of knowledge about emerging pathogens [12]. For instance, *E. coli* are commensal bacteria of the intestine of humans and animals but some pathogenic strains can cause moderate to severe gastrointestinal disease in humans. STEC is an emergent pathogen associated with foodborne diseases, although the risk of spontaneous abortion or preterm delivery in humans associated with STEC infection has not yet been evaluated.

Infections may cause early pregnancy loss (which occurs before 12 weeks of pregnancy), late pregnancy loss (which occurs between 12–20 weeks of gestation), or fetal death (which occurs during the third trimester) [24]. Giakoumelou et al. [25] performed a retrospective study of clinical cases to evaluate the role of infections during pregnancy. These authors concluded that there is evidence of infectious agents that affect pregnancy, as those cited before, and dismissed others including *Mycoplasma genitalium* and *Chlamydia Trachomatis,* for example. On the other hand, these authors confirmed the real ignorance about infectious agents that affect pregnancy and in which, physiological mechanisms are induced by infectious agents in fetal tissues [25].

## 3. Pathophysiology of Adverse Pregnancy Outcomes Caused by Infections

Feto-maternal interface is comprised of placental and fetal membranes (amnion and chorion). The feto-maternal interface may be divided into the fetal and the maternal side. In amongst the fetal side of the interface, the placenta and membranes, are allograft tissues. In this sense, fetal allograft contact the maternal blood through the syncytiotrophoblast, a specialized epithelial cell of the placenta that acts as an important component of the feto-maternal barrier. On the other hand, decidua is maternal remodeled uterine tissue in contact with the placenta and fetal membranes, where specialized immune cells such as macrophages and lymphocytes reside. A delicate and still not well-known mechanism in the feto-maternal interface occurs to allow tolerance of the allograft fetus during the pregnancy period. Once a microorganism makes contact with the host, there are several possible pathways that may be initiated, that may either individually or collectively promote adverse pregnancy outcomes. Bacteria or endotoxins such as lipopolysaccharide (LPS) are recognized by the innate immune system through receptors that recognize molecular patterns associated with pathogens. Toll-like receptors (TLRs) are signaling receptors that activate gene expression programs including the production of proinflammatory cytokines and type I interferons (INF). The TLR family includes TLR2 that mediates cellular responses to Gram positive organisms via glycolipids, peptidoglycans and lipoproteins. TLR4 is involved in activation of the innate immune system to exogenous ligands including LPS, which is abundant on the surface of pathogenic *E. coli.* When LPS binds its receptor, the nuclear factor of kappa B (NF-κB) becomes derepressed, and as a consequence, proinflammatory cytokines are expressed [26].

Host inflammatory signaling promotes the recruitment and activation of a variety of host immune cells. Resident phagocytes (neutrophils, macrophages), circulating inflammatory cells, antigen-presenting cells, and lymphocytes, in coordination have the function of identifying, antigen processing, and neutralizing invading pathogens. In addition, an effective innate immune response allows the proper recruitment and activation of specific immune cells necessary for a strong antibody response in order to promote the clearance of pathogens and/or to reduce the severity of illness upon reinfection [27]. However, innate immune cells such as neutrophils, may induce fatal disease in the mother and/or fetus instead to provide protection.

While inflammation of the uterine environment is essential for processes like implantation and placentation, its exacerbation may lead to complications such as premature delivery and pre-eclampsia. There is much evidence that inflammation in fetal tissues may impair long or short-term normal pregnancy. Histological infiltration of the tissue by neutrophils, macrophages and lymphocytes defines if inflammation is acute or chronic. There is a window of time between the cellular infiltrate and the molecular signals (induction of proinflammatory cytokines or adhesion molecules) that mediate this migration. It is often the case that this type of histological inflammation is ruled out when the systemic clinical symptoms such as inflammation, reddening, heat, pain and tumor are not found. Clinical evidence shows that most histological inflammations, including inflammation of chorioamniotic membranes, are subclinical [28].

## 4. STEC Infection May Be Responsible for Pregnancy Complications

To our knowledge, there are no reports of Stx effects during human pregnancy or described pregnancy complications associated with STEC infection. There are some reported cases of neonatal HUS caused by STEC transmission from mother to the newborn during delivery [29,30]. Also a few cases of STEC-mediated HUS during pregnancy have been reported [31,32,33].

Several studies in animal models have demonstrated that Stx2 causes spontaneous abortion and perinatal complications. It is possible that the effects observed may be a consequence of a direct action of Stx on the placental tissues. Even if most of the reported studies regarding Stx and pregnancy were referred to Stx2, we cannot exclude that similar events may be triggered by Stx1. With respect to this, both Stx1 and Stx2 have been reported to induce apoptosis in WISH cell line derived from human amniotic tissue [34] and Stx2 impairs migration and invasion in the cell line Swan71 used as a model of human trophoblasts [35].

Some authors have shown that Stx affects pregnancy in rodents. Burdet et al. [36] reported that a combination of Stx2 and LPS intraperitoneally injected in rats in the late stage of pregnancy produces preterm delivery of dead fetuses. An overproduction of nitric oxide (NO) and damage in the placenta prevented by aminoguanidine (AG), an inducible NO synthase inhibitor, demonstrated that NO plays an important role in placental toxicity and fetal mortality induced by Stx2 [37]. In addition, NO overproduction induces embryonic resorption in mice [38,39]. Moreover, NO may stimulate the activity of cyclooxygenase and cause an increase in prostaglandins (PGs) synthesis. In this sense, it has been reported that PGs, produced by uterine tissues in the surroundings of infection, cause cervical dilatation and uterine contractions leading to a premature delivery [40]. The production of PGs and expression of COX-2 protein were modulated by the NOS activity in Stx2-treated rats. Additionally, Stx2 may induce the production of tumor necrosis factor alpha (TNF-α) that renders the feto-maternal unit more susceptible to Stx2 through the stimulation of local PGs synthesis [41]. Many studies conducted in human and experimental animals determined that a correct balance of cytokines at the maternal-fetal interface is an essential requirement for correct placental development, and therefore, reproductive success [42,43]. Burdet et al. [41] reported that the combined action of AG and Etanercept, a recombinant human tumor necrosis factor receptor fusion protein that binds TNF-α, prevented preterm delivery. This data indicates that TNF-α may play a causal role in pregnancy loss consistent with previous studies of preterm delivery reported in humans [44].

Stx2 can also cause spontaneous abortion in mice by damaging trophoblasts and producing intrauterine hemorrhage, fibrin deposition, and neutrophil infiltration [45]. Abortion has been also demonstrated in rats, since morphological and histological damage in the uteroplacental unit as well as feto-maternal resorptions have been reported after sublethal dose of Stx2 in the early stage of pregnancy [46]. Stx2 binds to the microvasculature and decidual cells where a significant hypoxia and intrauterine growth restriction (IUGR) have been detected [47]. The detrimental effect of Stx2 was also enhanced by a local inflammation state rendering the cells more sensitive to the toxin as occurs in other target organs [48].

Both inflammation and hypoxia are adaptive mechanisms by which organisms respond to the disturbance of the function of an organ. Both mechanisms play an important role in pathological processes such as IUGR, spontaneous abortion, preeclampsia and preterm delivery [49]. Although, initially the uterine physiological environment for trophoblastic invasion is characterized as being hypoxic, it becomes highly oxygenated when the remodeling of the uterine spiral arteries is completed. Hypoxia is essential in normal fetal development for both vasculogenesis/angiogenesis, hematopoiesis, chondrogenesis and for feto-placental development in general [50]. However, excess of hypoxia leads to abnormalities in the development. Complications due to fetal hypoxia are one of the most important causes of fetal death [51]. It is known that hypoxia generates Reactive Oxygen Species (ROS) that can subsequently regulate the transcriptional and post-transcriptional response of hypoxia-responsive genes [52]. Therefore, the inhibition of ROS in hypoxic conditions inhibits some of the transcriptional responses triggered during this condition. Hypoxic conditions can also lead to a proinflammatory state [53]. Oxygen partial pressure regulates the inducible factor by hypoxia-1 (HIF-1). HIF-1 is composed of two subunits, HIF-1α sensitive to oxygen and HIF-1β constitutively expressed. HIF-1α is a labile protein against oxygen that stabilizes under hypoxic conditions [50]. This process is finely regulated by prolyl hydroxylases that hydroxylate HIF-1α under normoxic conditions and lead to its degradation by interaction with the tumor suppressor protein von-Hipple Lindau (p-VHL). Under conditions of hypoxia, HIF-α is not hydroxylated, does not bind to p-VHL and is translocated to the nucleus, activating the transcription of genes known as hypoxia response: vascular endothelial growth factor (VEGF), erythropoietin, transferrin, rust inducible nitric acid synthase, endothelin-1, among others [54]. Included within the genes regulated by HIF are those genes that regulate the process of angiogenesis, such as VEGF among others [55]. VEGF was described in rat placental tissues to be downregulated by Stx2 and we can speculate that inhibition of protein synthesis by the toxin may also alter HIF expression [47].

As previously was mentioned, several physiological changes occur in the mother during pregnancy, within which adequate cardiovascular adaptation (increased cardiac output) to provide uterine perfusion is necessary to meet fetal needs. The placenta needs adequate remodeling of the uterine arteries and vascularization to ensure the transport of nutrients and oxygen to the fetus. An impairment in the development of the placental vessels leads to fetal deterioration. The reduction in fetal blood flow may be due to a reduction in utero-placental blood flow or an abnormal villous structure at the feto-maternal-placental interface [56]. Moreover, the placental inflammatory state produces accumulation of immune cells that can lead to hypoxia by oxygen consumption of these cells added to the decrease in blood perfusion at the fetus-maternal-placental interface [57].

**Figure 1:**
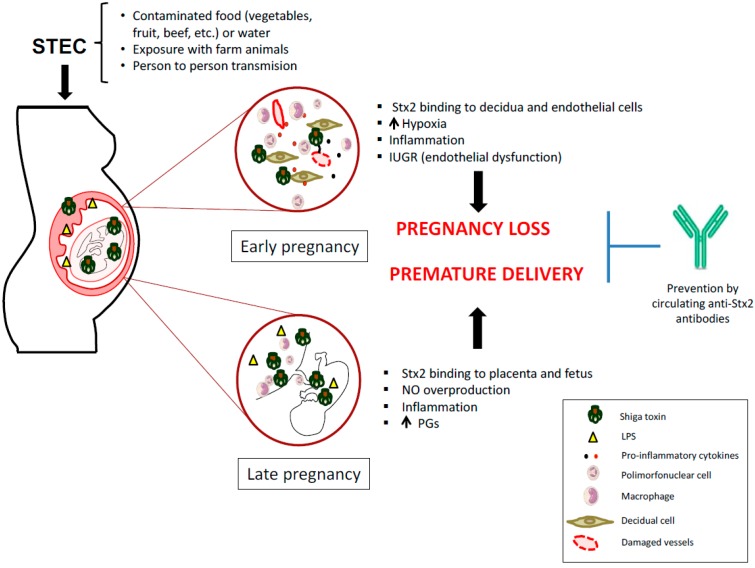
Mechanisms proposed for how Shiga toxin-producing *Escherichia coli* (STEC) infection may impair human pregnancy.

In this sense, symptomatic or asymptomatic STEC infections during early pregnancy may cause maternal or fetal damage mediated by Stx2. In consequence, STEC infection during pregnancy may cause maternal or fetal damage mediated by Stx2. These data support the hypothesis that similar events may occur during human pregnancy (Figure 1).

## 5. Strategies to Prevent STEC Infection During Pregnancy

Due to the high incidence of HUS in Argentina and the lack of a licensed vaccine or specific and effective therapy, primary prevention is fundamental to decrease the impact of HUS. One important challenge is to develop an effective, safe and nontoxic immunogen, also able to induce long-lasting, high-affinity antibodies that assure good neutralization capacity in serum. Epidemiological studies that refer to the prevalence of antibodies in children and in the adult population, showed that after the second decade of life, the anti-Stx antibody titers increases and then declines in the elderly [58]. These results agree with the incidence of HUS that mainly affects children. The study of two outbreaks of STEC infection associated the seropositivity of anti-Stx antibodies with protection to the development of symptoms associated with STEC, while the individuals seronegative for Stx had symptoms [59]. Prevalence studies of anti-Stx antibodies in Argentina show that 67% of healthy children have anti-Stx2 antibodies and 8% anti-Stx1 antibodies, while 86% of children with HUS had anti-Stx2 antibodies. In turn, it was also shown that there is more persistence of antibodies against the A subunit than the B subunit of Stx2 [60]. Consistent with these findings, a previous study reported anti-Stx2 seroreactivity in adults, generally refractory to HUS [61].

To date, there is still no specific treatment for HUS patients, leaving only the clinical management of support as an alternative. The prevention of infections by STEC and the development of HUS at the consumer level, lies in proper cooking of food, avoiding cross contamination (do not cut raw vegetables on the same table or with the same knife that cuts raw meat) and consuming pasteurized dairy products, to mention the most usual measures [62].

Epidemiological studies on the prevalence of anti-Stx antibodies in the population, reveals the importance of anti-Stx antibodies as a therapeutic strategy to prevent HUS. In the development of this strategy, several tests were carried out with anti-Stx monoclonal antibodies that have shown to be an effective therapeutic tool to protect cells and organs from the action of Stx2 in vitro and in vivo [63,64]. Additionally, a specific antibody against *E. coli* O157 LPS has been described in serum [33] as well as in breast milk of pregnant women with HUS [65], which may be advantageous for protecting the newborn against STEC infection. In animal models, it was demonstrated that maternal immunization against Stx2, protects dams from Stx2 mediated pregnancy loss. Additionally, immunized dams confer to the offspring, through lactation, protection against a lethal dose of Stx2 [66,67,68]. In this regard, maternal immunity against Stx may be important to protect pregnancy and the breastfeeding neonate (Figure 1). Recent advances that link maternal STEC infections with abortion and preterm labor suggest the importance of controlling STEC infections in pregnant women.

Further epidemiological studies about the prevalence of symptomatic or asymptomatic STEC infections in pregnant women and the correlation with adverse pregnancy outcomes will help to understand the role of STEC infections during pregnancy. Additionally, it will be relevant to demonstrate if anti-Stx antibodies in human milk have the ability to neutralize STEC infections in neonates in order to protect against HUS.
